# Effect of Cu-BTC-Modified Carbon Fiber on Interfacial and Mechanical Properties of Polyethylene Matrix Composites

**DOI:** 10.3390/molecules31152573

**Published:** 2026-07-23

**Authors:** Shuzhen Guo, Shanshan Xu, Yuhao Ma

**Affiliations:** 1 Shandong Fangda New Material Technology Co., Ltd., Zibo 255049, China; 2 School of Materials Science and Engineering, Shandong University of Technology, Zibo 255049, China

**Keywords:** polyethylene, carbon fiber, carboxylated, Cu-BTC

## Abstract

Carbon fiber (CF)-reinforced polyethylene (PE) composites have low density, outstanding corrosion resistance and good processability. These materials are widely promising for household appliances, automobiles and construction industries. Nevertheless, PE is a non-polar inert matrix with extremely low surface energy, leading to poor interfacial wettability and bonding force with CF. Interfacial debonding frequently occurs along with low load transfer efficiency, failing to meet the service requirements of high-performance structural components. In this study, CF was carboxylated with hydrogen peroxide, and Cu-BTC porous materials were in situ grown on the fiber surface to obtain modified CF (CF-Cu-BTC). The CF-Cu-BTC was then incorporated into a low-density polyethylene (LDPE) matrix. The MOF layer improves the interfacial compatibility and bonding force between the fibers and the matrix and enhances the overall mechanical properties and structural stability of the composites. The mechanical performance of CF composites is remarkably superior to that of pure LDPE. Compared with the pristine sample with a tensile strength of 11.04 MPa, the composite exhibits an enhanced tensile strength of 26.63 MPa, an increase of 141.20%. Scanning electron microscopy results confirm that no gaps exist between the CF and LDPE, verifying favorable interfacial compatibility. MOF-modified CF effectively improves the mechanical properties of resin-based composites. This study provides practical guidance for advanced composite applications.

## 1. Introduction

Polyethylene (PE) features excellent overall performance, superior chemical corrosion resistance and convenient molding processability [1,2,3]. It is widely applied in furniture manufacturing, automotive parts, civil pipelines and other fields, serving as a core basic material in the polymer material industry [4,5]. Nevertheless, neat PE has several intrinsic defects. Its tensile strength and thermal stability are insufficient, and the material creeps easily under high temperature [6,7]. These disadvantages hinder its mass application in high-end equipment and new energy components [8].

Doping inorganic fillers into a polyethylene matrix is a mainstream modification method to improve material properties [9,10,11]. The reinforcing phase can enhance mechanical performance via load transfer, stress dispersion and skeleton constraint. Common fillers include calcium carbonate, talc powder and silicon dioxide. Sibel Tuna et al. [12] prepared wollastonite-filled PE composites and found that the filler increased the flexural modulus and thermal stability of the composites, while poor interfacial compatibility led to a decreased elongation at break. Gill et al. [13] prepared silanized nano-silica/XLPE composites via two-step blending with DCP; the filler improved thermal, UV and flame resistance, yet overloading caused agglomeration and reduced the dielectric property. Compared with particulate fillers, fibrous fillers possess high specific strength and specific modulus, serving as vital reinforcing components for polymer composites [14,15,16]. Typical examples include glass fiber, quartz fiber and CF. Zhang et al. [17] fabricated SCF/PEI composites and investigated their tensile creep, finding that higher fiber loading and fiber thermal treatment improved creep resistance. Compared with glass fiber and other traditional fillers, CF greatly improves PE’s mechanical and thermal performances and has become a key filler for PE modification [18,19,20].

However, there is a significant polarity difference between CF and the PE matrix in composites, resulting in poor interfacial wettability and compatibility [21,22]. This easily causes fiber agglomeration and internal pore defects, degrading the overall performance of composites [23]. Relevant research including CF carboxylation and surface sizing has achieved certain improvements. Xiao et al. [24] prepared an epoxy-based sizing agent to modify CF for CF/PA6 composites and found that fiber surface modification raised fiber roughness and active groups, improving composite tensile and impact strength via enhanced interfacial bonding. However, these methods tend to etch the fiber surface, damage the fiber structure and degrade its intrinsic mechanical performance. Meanwhile, conventional epoxy sizing agents are designed for thermosetting resins and exhibit poor compatibility with non-polar LDPE. Accordingly, a mild interfacial modification strategy is required to improve CF/LDPE interfacial bonding without damaging fiber structures.

As a new type of porous material assembled from metal centers and organic ligands, metal–organic frameworks (MOFs) have attracted extensive research interest in recent years owing to their abundant pore channels and functional groups [25,26]. Among them, Cu-BTC is a representative MOF that can be synthesized under relatively mild conditions from readily available precursors. Compared with simple oxidation or conventional sizing/compatibilizer modification, Cu-BTC can construct an interphase layer on pre-oxidized carbon fiber surfaces. This structure strengthens physical adhesion and mechanical interlocking between carbon fibers and the non-polar LDPE matrix and improves the efficiency of interfacial stress transfer. Previous studies have reported MOF-modified carbon fibers for improving the interfacial performance of polymer composites [27,28]; their application in non-polar polyethylene matrices, especially LDPE, remains limited. Therefore, Cu-BTC is used to modify carbon fibers in this work. This treatment alleviates the interface mismatch between CF and LDPE and improves fiber reinforcing efficiency in non-polar thermoplastic matrices.

In this study, CF was carboxylated using hydrogen peroxide, and Cu-BTC was subsequently grown in situ on the fiber surface. The obtained CF-Cu-BTC was then melt-blended with LDPE to prepare CF-Cu-BTC/LDPE composites. A CF/LDPE-20 control sample was additionally fabricated to differentiate the intrinsic reinforcement of the rigid CF skeleton from the performance enhancement induced by Cu-BTC interfacial modification. In situ grown Cu-BTC forms a modified interphase on CF. It strengthens CF/LDPE interfacial adhesion and enables efficient stress transfer during tension and bending. The effects of different CF contents on material properties were investigated. At the optimal filling ratio, the tensile strength of the composite reaches 26.63 MPa, which is 141.2% higher than that of pure LDPE. The results prove that modified CF exerts a favorable reinforcement effect, enhances interfacial bonding force and improves the mechanical properties of composites. This research provides a theoretical reference and technical support for the application of high-performance CF/LDPE composites in high-end structural components.

## 2. Results and Discussion

### 2.1. Structural Characterization of CF-Cu-BTC/LDPE Composites

**Figure 1:**
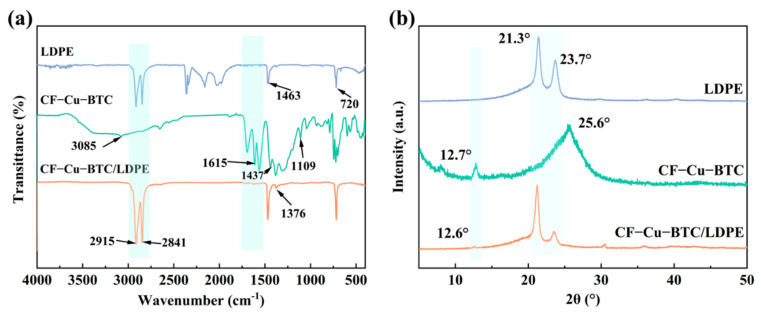
Structural characterization of neat LDPE, CF-Cu-BTC and CF-Cu-BTC/LDPE composite: (**a**) FTIR spectra and (**b**) XRD patterns.

The FTIR spectra of neat LDPE, CF-Cu-BTC and CF-Cu-BTC/LDPE composites are shown in Figure 1a. For neat LDPE, the characteristic absorption peaks at 2915 and 2841 cm^−1^ correspond to the asymmetric and symmetric stretching vibrations of –CH_2_– groups, respectively [29]. The absorption band at 1463 cm^−1^ is attributed to the bending vibration of –CH_2_– groups, while the peak around 720 cm^−1^ is assigned to the rocking vibration of long methylene sequences in polyethylene. These characteristic peaks confirm the typical chemical structure of LDPE. For CF-Cu-BTC, the broad absorption band around 3085 cm^−1^ is associated with the stretching vibration of –OH groups, indicating that oxygen-containing functional groups were introduced onto the CF surface after H_2_O_2_ oxidation [30]. The absorption band at 1615 cm^−1^ can be assigned to the asymmetric stretching vibration of coordinated carboxylate groups and the skeletal vibration of aromatic rings in BTC ligands. The peak at 1437 cm^−1^ is related to the symmetric stretching vibration of carboxylate groups, suggesting the coordination interaction between BTC ligands and Cu^2+^ ions [31]. In addition, the absorption band at 1109 cm^−1^ is attributed to C–O stretching vibration. These characteristic bands indicate the successful construction of BTC-related coordination structures on the CF surface. For the CF-Cu-BTC/LDPE composite, the characteristic absorption peaks of LDPE are still retained, including the –CH_2_– stretching vibrations at 2915 and 2841 cm^−1^, the –CH_2_– bending vibration at 1463 cm^−1^ and the rocking vibration around 720 cm^−1^. In addition, weak absorption bands around 1702 and 1615 cm^−1^ can be observed in the composite spectrum. The band at 1702 cm^−1^ may be related to the C=O stretching vibration of carboxyl groups, while the band at 1615 cm^−1^ may be associated with the vibration of coordinated carboxylate groups and BTC aromatic skeleton. These weak Cu-BTC-related signals suggest the presence of CF-Cu-BTC in the LDPE matrix. However, their intensities are relatively low because of the dominant LDPE phase and possible overlap with matrix-related absorption bands.

The XRD patterns of LDPE, CF-Cu-BTC and CF-Cu-BTC/LDPE composite are shown in Figure 1b. Neat LDPE exhibits two characteristic diffraction peaks at 21.3° and 23.7°, which can be assigned to the (110) and (200) crystal planes of polyethylene, respectively [32]. These peaks indicate the typical crystalline structure of LDPE. For CF-Cu-BTC, an obvious diffraction peak appears at 12.7°, which can be associated with the low-angle characteristic diffraction of Cu-BTC [33], indicating the successful formation of Cu-BTC on the CF surface. In addition, the broad diffraction peak at around 25.6° can be attributed to the graphitic structure of carbon fibers. After incorporation into the LDPE matrix, the CF-Cu-BTC/LDPE composite still retains the main diffraction peaks of LDPE, suggesting that the crystalline structure of LDPE is largely maintained after melt blending. Meanwhile, a weak Cu-BTC-related diffraction peak can still be observed at around 12.6° in the composite, further supporting the presence of CF-Cu-BTC in the LDPE matrix. The slight shift and reduced intensity of this peak may be attributed to the dilution effect of the LDPE matrix, partial peak overlap and the relatively low exposed amount of Cu-BTC in the composite system. Overall, the XRD results further confirm the successful construction of CF-Cu-BTC and indicate that the introduction of CF-Cu-BTC does not significantly change the main crystalline structure of LDPE.

### 2.2. Thermal Stability and Crystallization Behavior of CF-Cu-BTC/LDPE Composites

**Figure 2:**
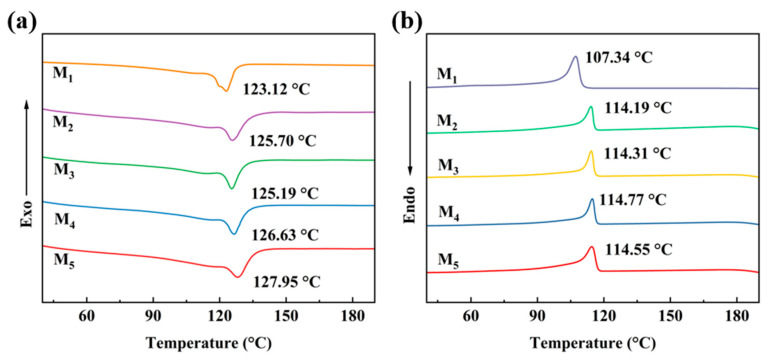
DSC curves of LDPE and CF-Cu-BTC/LDPE composites: (**a**) heating curves and (**b**) cooling curves.

Figure 2 displays the DSC heating and cooling curves of pure LDPE and CF-Cu-BTC/LDPE composites. The corresponding thermal parameters are listed in Table S1. As shown in Figure 2a, pure LDPE presents a melting peak at 123.12 °C. After adding CF-Cu-BTC, the melting peak temperatures of M_2_, M_3_, M_4_ and M_5_ are 125.70 °C, 125.19 °C, 126.63 °C and 127.95 °C, respectively. The melting temperature only changes slightly. This indicates that the incorporation of CF-Cu-BTC has little effect on the melting characteristics of LDPE. The main crystal structure of LDPE remains stable after melt blending. The cooling curves in Figure 2b reveal that CF-Cu-BTC affects the crystallization behavior of LDPE. Pure LDPE shows a crystallization peak at 107.34 °C. In comparison, the crystallization temperatures of M_2_, M_3_, M_4_ and M_5_ increase to 114.19 °C, 114.31 °C, 114.77 °C and 114.55 °C, respectively. The increased crystallization temperature demonstrates that CF-Cu-BTC promotes LDPE crystallization during the cooling process. This phenomenon follows the general heterogeneous nucleation mechanism of filler/polyethylene systems [34]. This can be attributed to the heterogeneous nucleation effect of CF and Cu-BTC particles, which provide additional nucleation sites for LDPE molecular chains and reduce the nucleation barrier, allowing crystallization to occur at a higher temperature. The crystallinity of LDPE was calculated according to the following equation:

(1) $$X_{c}=\frac{\Delta H_{m}}{\left(1-\varphi \right)\Delta H_{m}^{0}}\times 100\%$$

where $\Delta H_{m}$ is the melting enthalpy obtained from DSC, φ is the mass fraction of CF-Cu-BTC, and $\Delta H_{m}^{0}$ is the melting enthalpy of 100% crystalline PE, taken as 293 J·g^−1^ [35,36]. The X_c_ value of neat LDPE is 49.59%. After the incorporation of CF-Cu-BTC, the X_c_ values of M_2_, M_3_, M_4_ and M_5_ are 37.33%, 39.79%, 38.49% and 32.28%, respectively. Compared with neat LDPE, the crystallinity of all composites decreases, indicating that the rigid CF-Cu-BTC phase restricts the mobility and regular arrangement of LDPE molecular chains. It should be noted that M_3_ shows a slightly higher crystallinity than M_2_ and M_4_, which may be attributed to the heterogeneous nucleation effect at a moderate filler content. However, when the CF-Cu-BTC content further increases, the interfacial confinement and filler restriction become more pronounced, inhibiting crystal growth and perfection. Therefore, CF-Cu-BTC plays a dual role in the crystallization behavior of LDPE: it promotes crystallization nucleation during cooling, while excessive filler loading restricts LDPE chain mobility and reduces the final crystallinity.

**Figure 3:**
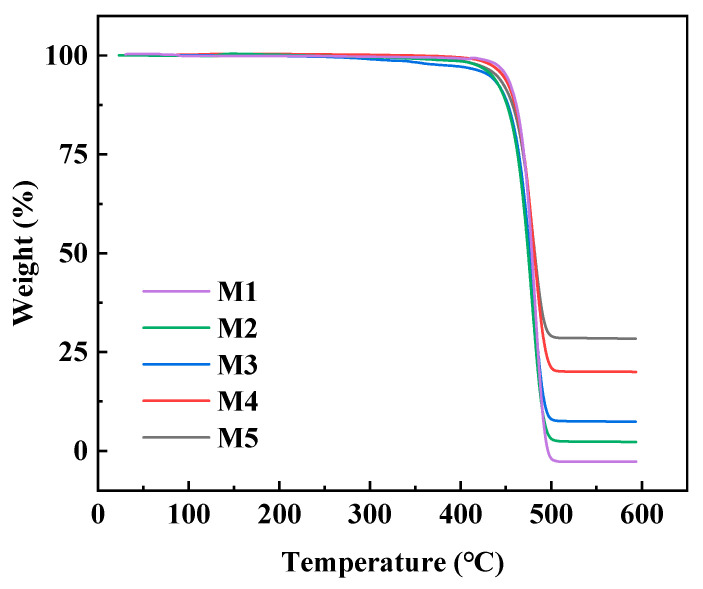
TG curves of LDPE and CF-Cu-BTC/LDPE composites with different CF-Cu-BTC contents.

The thermal stability of LDPE and CF-Cu-BTC/LDPE composites was evaluated by TG, as shown in Figure 3, and the corresponding thermal decomposition parameters are summarized in Table S2. Neat LDPE exhibits a typical one-step thermal degradation behavior, which is consistent with the thermal decomposition characteristics of polyethylene-based materials reported in previous studies [36,37]. The main weight loss occurs in the range of approximately 450–510 °C, which is attributed to the thermal decomposition of the polyethylene molecular chains. After degradation, almost no residual mass remains, indicating the complete decomposition of the LDPE matrix under nitrogen atmosphere. After the incorporation of CF-Cu-BTC, the composite shows a similar main degradation process, suggesting that the thermal decomposition behavior is still dominated by the LDPE matrix. The T_onset_ values of M_2_, M_3_, M_4_ and M_5_ are 453.76, 456.10, 458.10 and 456.84 °C, respectively, while their T_max_ values are 478.47, 479.76, 480.90 and 479.91 °C, respectively. Compared with neat LDPE, the degradation temperatures of the composites show only slight changes. This indicates that the introduction of CF-Cu-BTC does not significantly alter the main thermal decomposition behavior of LDPE. The slight decrease in T_5%_ and T_max_ for some composites may be related to the presence of Cu-BTC and Cu-containing species, which can affect the thermal degradation process of LDPE to some extent [31]. Notably, the residual mass increases with increasing CF-Cu-BTC content. The residue increases from nearly 0% for neat LDPE to 2.29%, 7.37%, 19.99% and 28.43% for M_2_, M_3_, M_4_ and M_5_, respectively. This increase is mainly attributed to the thermally stable carbon fibers and inorganic Cu-containing residues derived from Cu-BTC [38]. In particular, the residual masses of M4 and M5 are close to their designed CF-Cu-BTC contents, further supporting the successful incorporation of CF-Cu-BTC into the LDPE matrix.

### 2.3. Morphology and Elemental Analysis

**Figure 4:**
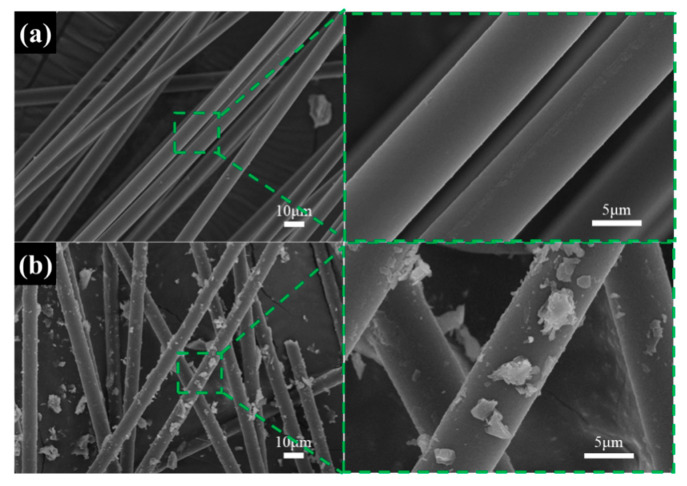
SEM images of CF before and after surface modification: (**a**) pristine CF and (**b**) CF-Cu-BTC-modified CF.

The surface morphologies of pristine CF and CF-Cu-BTC-modified CF are shown in Figure 4. As shown in Figure 4a, the pristine CF exhibits relatively smooth and clean surfaces, with only slight longitudinal grooves along the fiber axis. No obvious particles or coating layers can be observed on the fiber surface, indicating the inert and smooth nature of the untreated CF. After H_2_O_2_ oxidation and in situ growth of Cu-BTC, obvious morphological changes can be observed on the CF surface. As shown in Figure 4b, numerous particle-like and sheet-like structures are distributed on the surface of the modified CF. Compared with pristine CF, the surface of CF-Cu-BTC becomes much rougher, indicating that Cu-BTC was successfully introduced onto the CF surface. The high-magnification SEM image further confirms that the modified fiber surface is covered by discrete Cu-BTC particles rather than remaining smooth. In situ formed Cu-BTC constructs a modified interphase layer on CF, which improves the interfacial bonding strength and stress transfer efficiency of composites. Therefore, the SEM results provide direct morphological evidence for the construction of the Cu-BTC interfacial layer on CF.

**Figure 5:**
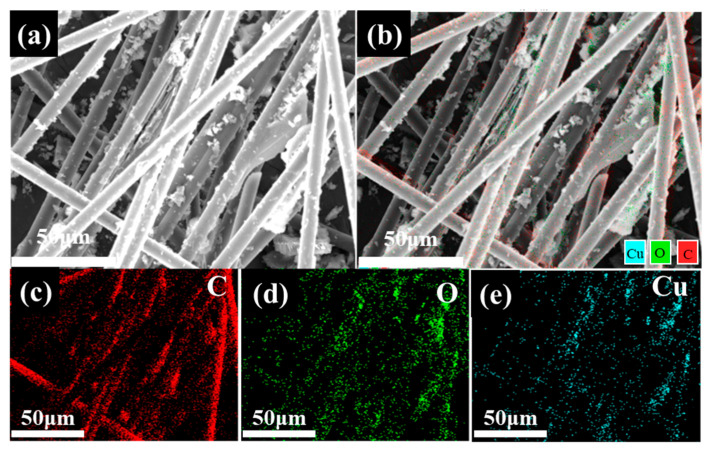
EDS elemental mapping of CF-Cu-BTC-modified CF: (**a**) SEM image, (**b**) layered elemental mapping, (**c**) C mapping, (**d**) O mapping and (**e**) Cu mapping.

EDS elemental mapping analysis is shown in Figure 5. The SEM image in Figure 5a shows that the modified CF are covered with obvious particulate structures. The layered elemental mapping image in Figure 5b further reveals the distribution of C, O and Cu on the fiber surface. As shown in Figure 5c, the C signal is mainly derived from the CF substrate. The O signal in Figure 5d can be attributed to the oxygen-containing groups introduced by H_2_O_2_ oxidation and the carboxylate groups of BTC ligands. More importantly, the Cu signal shown in Figure 5e is clearly detected on the modified CF, indicating the successful introduction of Cu-containing species. The presence of both O and Cu confirms that Cu-BTC was successfully constructed on the CF surface. Combined with the SEM observations, the EDS results demonstrate that the in situ growth process formed a rough Cu-BTC coating layer on the CF. This Cu-containing modified interphase layer provides structural evidence for the successful construction of CF-Cu-BTC, which is beneficial for improving the interfacial bonding force and stress transfer efficiency between CF and the LDPE matrix.

**Figure 6:**
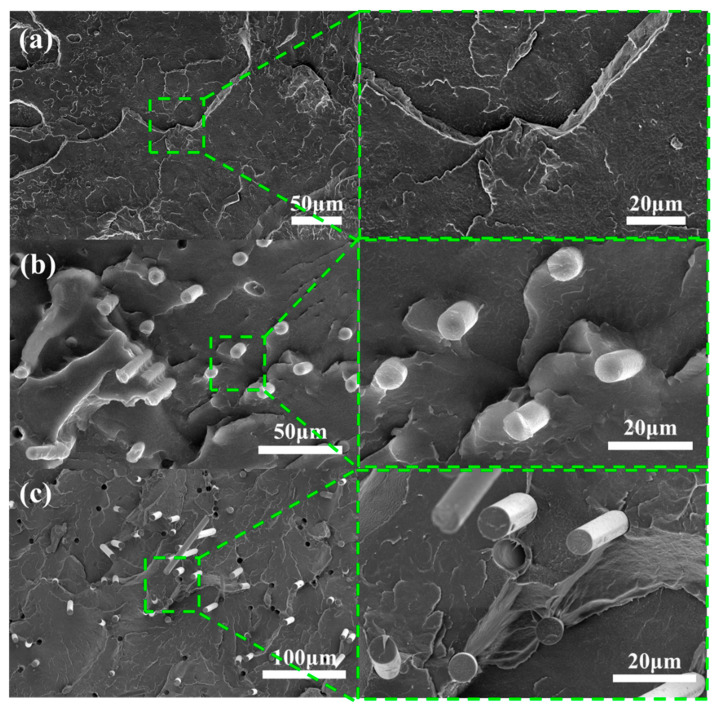
SEM images of liquid-nitrogen cryo-fractured surfaces of (**a**) neat LDPE, (**b**) M_4_ CF-Cu-BTC/LDPE composite and (**c**) CF/LDPE-20 composite.

Figure 6 shows the SEM micrographs of liquid-nitrogen cryo-fractured surfaces of neat LDPE, M_4_ CF-Cu-BTC/LDPE composite and CF/LDPE-20 control composite. As shown in Figure 6a, neat LDPE exhibits a continuous matrix morphology with typical plastic deformation and tearing features, and no fiber-like reinforcing phase can be observed. This indicates that the fracture morphology is mainly governed by the deformation of the LDPE matrix itself. After the incorporation of CF-Cu-BTC, a significantly different morphology can be observed for the M_4_ composite, as shown in Figure 6b. The modified fibers are distributed in the LDPE matrix, and some fibers are partially embedded in the matrix. The surrounding LDPE matrix is attached to parts of the fiber surface, suggesting improved interfacial interaction between CF-Cu-BTC and LDPE. This improved fiber/matrix contact can be attributed to the Cu-BTC-modified interphase layer on the CF surface, which facilitates stress transfer from the LDPE matrix to the rigid CF during mechanical loading. For comparison, the CF/LDPE-20 control composite containing untreated CF is shown in Figure 6c. Compared with M_4_, more obvious fiber pull-out, interfacial gaps and voids can be observed in the control sample. In addition, the exposed untreated fibers show relatively smooth surfaces and limited matrix adhesion, indicating weaker interfacial interaction between untreated CF and LDPE. These morphological differences demonstrate that the Cu-BTC modification improves the fiber/matrix interfacial contact and contributes to more efficient stress transfer in CF-Cu-BTC/LDPE composites.

### 2.4. Mechanical Properties of CF-Cu-BTC/LDPE Composites

**Figure 7:**
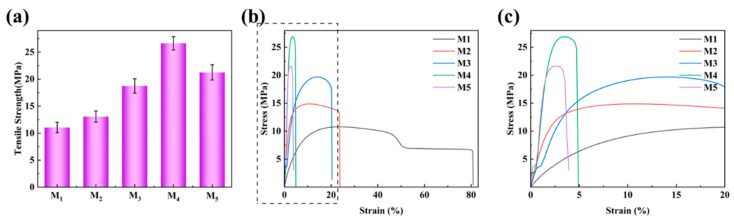
Tensile properties of LDPE and CF-Cu-BTC/LDPE composites: (**a**) tensile strength, (**b**) complete tensile stress–strain curves and (**c**) enlarged stress–strain curves in the strain range of 0–20%. The dashed box in (**b**) indicates the enlarged strain region shown in (**c**).

The tensile properties of neat LDPE and CF-Cu-BTC/LDPE composites are shown in Figure 7. As shown in Figure 7a, neat LDPE exhibited a tensile strength of 11.04 MPa. After the incorporation of CF-Cu-BTC, the tensile strength of the composites increased gradually with increasing filler content. The highest tensile strength was obtained for M_4_, reaching 26.63 MPa, which was approximately 141.20% higher than that of neat LDPE. This improvement can be attributed to the synergistic effect of the rigid CF skeleton and the Cu-BTC-modified interphase layer. The rigid CF can bear part of the external load, while the Cu-BTC interphase improves the fiber/matrix interaction and facilitates stress transfer from the LDPE matrix to CF [39,40]. Under tensile loading, the optimized interphase enabled more efficient stress transfer from the LDPE matrix to the rigid high-strength CF, thereby enhancing the tensile strength of the composites. However, when the CF-Cu-BTC content further increased to 30 wt%, the tensile strength decreased to 21.25 MPa. Although this value was still higher than that of neat LDPE, it was lower than that of M_4_. This decline is related to the excessive CF loading, which tends to cause fiber aggregation, inferior dispersion and local stress concentration within the LDPE matrix [19]. These defects weaken the effective stress transfer at the interface and reduce the reinforcing efficiency of the CF-Cu-BTC. The complete stress–strain curves in Figure 7b and the enlarged curves in Figure 7c illustrate the evolution of tensile deformation behavior with increasing CF-Cu-BTC loading. Neat LDPE displays typical ductile behavior with low tensile stress and high elongation at break. Incorporating CF-Cu-BTC greatly raises composite stress levels yet lowers elongation, as rigid CF-Cu-BTC limits LDPE chain motion and plastic deformation, boosting stiffness at the cost of ductility. Under tension, CF skeletons share external loads, and the Cu-BTC interphase strengthens fiber-matrix bonding to facilitate efficient stress transfer from matrix to fiber, accounting for elevated strength and reduced ductility. Sample M_4_ achieves the maximum tensile stress, proving 20 wt% CF-Cu-BTC strikes the optimal balance between load-bearing reinforcement and interfacial stress transfer. In summary, moderate CF-Cu-BTC addition effectively enhances the tensile strength and stiffness of LDPE composites; excessive filler causes fiber aggregation and local stress concentration, weakening reinforcing efficiency.

The tensile modulus and elongation at break are presented in Figure S1. The tensile modulus of neat LDPE is 220 MPa, which rises to 860 MPa for sample M_5_, demonstrating that the CF-Cu-BTC filler can effectively enhance the stiffness of LDPE composites. In contrast, the elongation at break declines from 71.58% to 4.4% with the increase in CF-Cu-BTC loading. This observation reveals that rigid fibers constrain the tensile deformation of the LDPE matrix, reducing the ductility of the material and inducing a shift toward brittle fracture characteristics.

**Figure 8:**
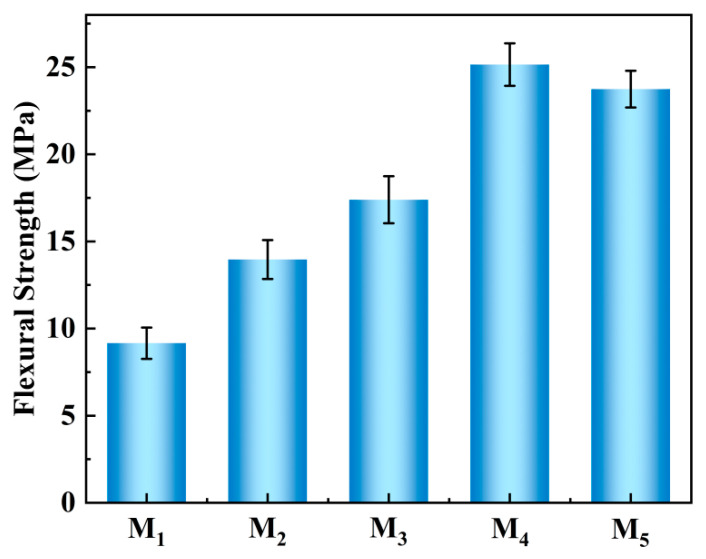
Flexural strength of LDPE and CF-Cu-BTC/LDPE composites.

The flexural strength of LDPE and CF-Cu-BTC/LDPE composites is shown in Figure 8. Neat LDPE exhibited a flexural strength of 9.16 MPa. After the incorporation of CF-Cu-BTC, the flexural strength increased significantly with increasing filler content. The flexural strength of M_2_ and M_3_ increased to 13.96 and 17.39 MPa, respectively, indicating that the rigid CF-Cu-BTC phase effectively enhanced the bending resistance of the LDPE matrix. Among all the samples, M_4_ exhibited the highest flexural strength of 25.15 MPa, which was 174.6% higher than that of neat LDPE. This improvement can be attributed to the synergistic effect of the rigid CF skeleton and the Cu-BTC-modified interphase layer. During bending deformation, the CF skeleton can act as a load-bearing phase to resist external stress, while the Cu-BTC-modified interphase layer improves fiber/matrix interaction and promotes stress transfer from the LDPE matrix to CF. Therefore, the applied bending stress can be more effectively transferred across the fiber/matrix interface, resulting in improved flexural strength. When the CF-Cu-BTC content further increased to 30 wt%, the flexural strength decreased slightly to 23.74 MPa. These defects weaken the effective stress transfer and reduce the reinforcing efficiency of CF-Cu-BTC. Nevertheless, the flexural strength of M_5_ was still much higher than that of neat LDPE, confirming the reinforcing effect of CF-Cu-BTC in the LDPE matrix.

**Figure 9:**
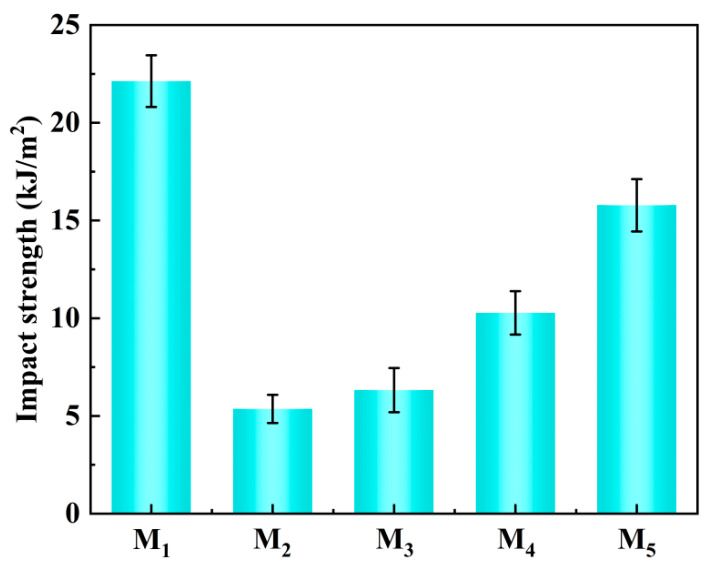
Impact strength of LDPE and CF-Cu-BTC/LDPE composites.

The impact strength of LDPE and CF-Cu-BTC/LDPE composites is shown in Figure 9. Neat LDPE exhibited the highest impact strength of 22.13 kJ·m^−2^, indicating its relatively good toughness and plastic deformation capability. After the incorporation of CF-Cu-BTC, the impact strength decreased sharply to 5.36 kJ·m^−2^ for M_2_. This decrease can be attributed to the introduction of rigid CF, which restricted the mobility of LDPE molecular chains and reduced the plastic deformation ability of the matrix under impact loading [41]. In addition, at low filler loading, the number of fibers is insufficient to form effective crack-deflection or energy-dissipation pathways, while the fiber ends and local interfacial regions may act as stress concentration sites, leading to premature crack initiation and propagation. With further increasing CF-Cu-BTC content, the impact strength gradually increased from 6.32 kJ·m^−2^ for M_3_ to 10.27 kJ·m^−2^ for M_4_ and 15.78 kJ·m^−2^ for M_5_. This recovery suggests that higher CF-Cu-BTC loading introduces additional energy-dissipation mechanisms, including crack deflection, fiber pull-out, interfacial friction and the formation of more tortuous crack propagation paths [42,43]. Meanwhile, the Cu-BTC-modified interphase layer can improve the fiber/matrix interaction to some extent, which helps delay rapid interfacial debonding and crack propagation during impact fractures. However, the impact strength of all CF-Cu-BTC/LDPE composites remained lower than that of neat LDPE. This indicates that the incorporation of rigid CF improves the tensile and flexural properties of LDPE composites but inevitably sacrifices part of the impact toughness. Therefore, the mechanical performance of CF-Cu-BTC/LDPE composites reflects a balance between stiffness/strength enhancement and toughness retention.

**Figure 10:**
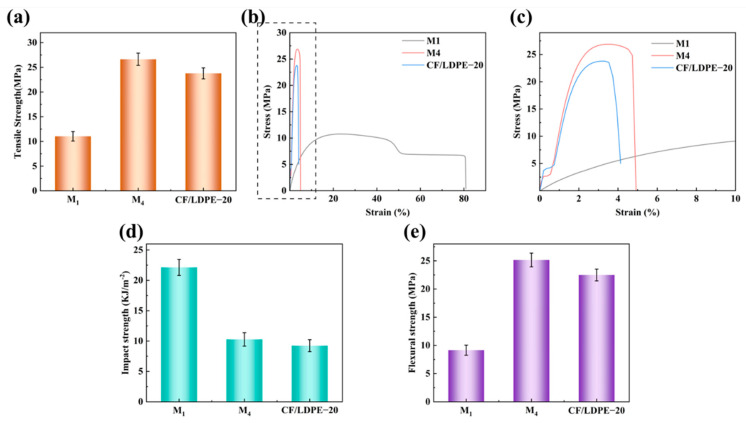
Comparison of mechanical properties of neat LDPE, M_4_ CF-Cu-BTC/LDPE composite and CF/LDPE-20 control composite: (**a**) tensile strength, (**b**) tensile stress–strain curves, (**c**) enlarged tensile stress–strain curves in the strain range of 0–10%, (**d**) impact strength and (**e**) flexural strength. The dashed box in (**b**) indicates the enlarged strain region shown in (**c**).

To further clarify the contribution of Cu-BTC modification, the mechanical properties of neat LDPE, M_4_ CF-Cu-BTC/LDPE composite and CF/LDPE-20 control composite were compared, as shown in Figure 10. The CF/LDPE-20 control sample contained the same filler content as M_4_, but the carbon fibers were not modified by Cu-BTC. Compared with neat LDPE, CF/LDPE-20 exhibited improved tensile and flexural strengths of 23.78 and 22.09 MPa, respectively, confirming the reinforcing effect of rigid CF in the LDPE matrix. However, M_4_ showed higher tensile and flexural strengths of 26.63 and 25.15 MPa, respectively, which were approximately 12.0% and 13.9% higher than those of CF/LDPE-20 at the same filler content. This result indicates that the mechanical enhancement of M_4_ cannot be attributed solely to the addition of CF; the Cu-BTC-modified interphase layer further improves the reinforcing efficiency of CF by enhancing fiber/matrix interaction and stress transfer efficiency. The impact strength shows a similar comparison between M_4_ and CF/LDPE-20. The impact strength of CF/LDPE-20 was 9.24 kJ·m^−2^, while that of M_4_ increased to 10.27 kJ·m^−2^. Although both composites showed a lower impact strength than neat LDPE due to the introduction of rigid fibers, the slightly higher impact strength of M_4_ suggests that the Cu-BTC-modified interphase layer may help retard crack propagation and improve energy dissipation to some extent. Overall, the comparison with the CF/LDPE-20 control sample further confirms that the improved mechanical performance of M_4_ results from the synergistic effect of the rigid CF skeleton and the Cu-BTC-modified interphase layer.

**Figure 11:**
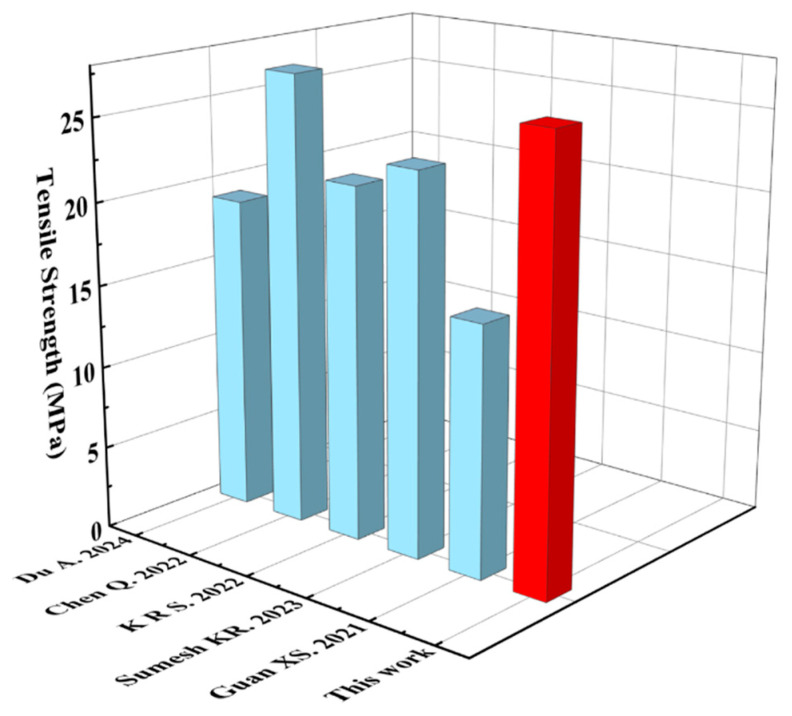
Comparison of tensile strength between this work and previously reported CF/PE-based composites [1,21,44,45,46].

To demonstrate the reinforcing effect of interfacial modification in the CF-Cu-BTC/LDPE composite system, the tensile strength of Sample M_4_ was compared with that of CF/PE-based composites reported in the existing literature, as shown in Figure 11 [1,21,44,45,46]. Sample M_4_ reaches a tensile strength of 26.63 MPa and exhibits favorable mechanical enhancement compared with most reported CF/PE composites. Detailed information is shown in Table S3.

### 2.5. Interfacial Reinforcement Mechanism

**Figure 12:**
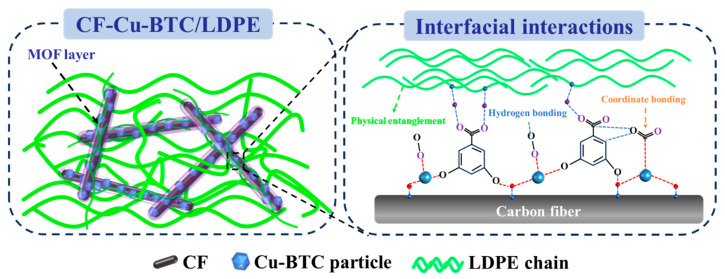
Schematic illustration of the interfacial reinforcement mechanism of CF-Cu-BTC/LDPE composites.

The interfacial reinforcement mechanism of CF-Cu-BTC/LDPE composites is illustrated in Figure 12. After hydrogen peroxide oxidation, oxygen-containing functional groups are introduced onto CF surfaces, which serve as active sites for the in situ growth of Cu-BTC. The in situ fabricated Cu-BTC constructs a modified interphase layer on CF and improves the interfacial bonding force between CF and the LDPE matrix, thereby elevating the mechanical properties of the resultant composites. Under tensile and flexural loads, the rigid CF skeleton can bear part of the external stress, while the optimized Cu-BTC interphase facilitates efficient stress transfer from the LDPE matrix to the high-strength CF. Compared with the CF/LDPE-20 control sample, the M_4_ composite exhibits improved mechanical properties and more favorable interfacial morphology, indicating that Cu-BTC modification further enhances the reinforcing efficiency of CF. Accordingly, specimen M_4_ delivers the optimal tensile and flexural strengths among all formulated samples. Nevertheless, excessive incorporation of CF-Cu-BTC tends to trigger fiber agglomeration and local stress concentration, deteriorating the reinforcing efficiency. In summary, the improved mechanical performance of composites originates from the synergistic reinforcement effect derived from the rigid CF skeleton, Cu-BTC interphase layer and efficient stress transfer at the fiber-matrix interface.

## 3. Experimental

### 3.1. Materials

Low-density polyethylene (LDPE, Grade 2650), China Petroleum & Chemical Corporation (Sinopec), Beijing, China; Carbon fiber (T700SC, length: 2 cm) was purchased from Shenzhen Turing New Materials Co., Ltd., Shenzhen, China; Antioxidant 168, Dongguan Dinghai Plastic Chemical Co., Ltd., Shenzhen, China. Cu(NO_3_)_2_·3H_2_O (99% purity) and 1,3,5-benzenetricarboxylic acid (H_3_BTC, 98% purity) were purchased from Saan Chemical Technology (Shanghai) Co., Ltd., Shanghai, China. Ethanol (EtOH, 99% purity) and hydrogen peroxide were purchased from Shanghai Aladdin BioChem Technology Co., Ltd., Shanghai, China. All chemicals and solvents were used as received, if not mentioned otherwise.

### 3.2. Synthesis of Modified CF and CF-Cu-BTC

**Figure 13:**
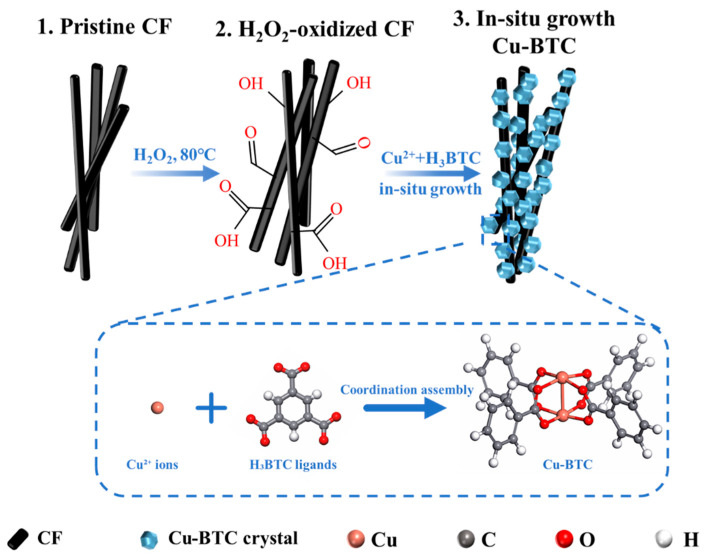
Schematic illustration of H_2_O_2_ oxidation and in situ growth of Cu-BTC on CF.

The synthesis method of CF-MOF was slightly adjusted based on the preparation process of modified CF. A total of 200 g desized CF was immersed in 50 mL hydrogen peroxide solution with a mass fraction of 30% and reacted at a constant temperature of 80 °C. After the reaction, the sample was taken out and thoroughly rinsed with deionized water and then dried at 80 °C for 12 h to obtain carboxylated modified CF. A total of 50 g modified CF, 0.875 g copper nitrate trihydrate and 0.42 g trimesic acid were added into 24 mL of an equal-volume water–ethanol mixed solution in a 100 mL round-bottom flask. The mixture was reacted at a constant temperature of 120 °C under stirring for 12 h. The sample was taken out and thoroughly washed with ethanol three times to remove residual precursors and then dried in a vacuum oven at 100 °C for 10 h to obtain a CF-Cu-BTC sample for subsequent use. The surface modification process of CF is shown in Figure 13.

### 3.3. Fabrication of CF-Cu-BTC/LDPE Composite

CF-Cu-BTC/LDPE composites were fabricated via melt blending of modified CF (length: 2 cm) and low-density polyethylene (LDPE). Firstly, LDPE pellets and modified CF were weighed according to a preset mass ratio, uniformly mixed by a mixer, and then fed into a twin-screw extruder for melt blending. The temperature of each extruder zone was set as follows: Zone 1 at 150 °C, Zone 2 at 160 °C, Zones 3–5 at 180 °C. Sufficient melting of LDPE and homogeneous dispersion of modified CF in the matrix were achieved. After melt blending, the extruded materials were cooled and shaped in a water tank, pelletized by a cutter, and finally dried in an oven at 100 °C for 8 h to obtain CF-Cu-BTC/LDPE composite pellets. For comparison, a control composite consisting of 20 wt% untreated carbon fiber and 80 wt% LDPE was fabricated via the identical melt compounding process, which was designated CF/LDPE-20. All pellets were injection-molded at 180 °C and demolded after 30 s of water cooling to obtain standard test specimens for subsequent characterizations. The blending formulas of all groups are listed in Table 1.

### 3.4. Characterization

Fourier transform infrared (FT-IR) spectra were recorded using a Bruker TENSOR II spectrometer in ATR mode to characterize LDPE and CF-Cu-BTC/LDPE composites. The morphology of the samples was observed using a Gemini SEM 500 scanning electron microscope (SEM), and an elemental mapping of the CF-Cu-BTC/LDPE composites was obtained with an integrated energy-dispersive X-ray spectrometer (EDS). Thermogravimetric analysis (TG) was conducted using a TASDT Q600 analyzer. Samples of 10–20 mg LDPE and CF-Cu-BTC/LDPE composites were heated from 40 °C to 600 °C at 10 °C/min under a nitrogen flow of 100 mL/min to record weight loss. Differential scanning calorimetry (DSC) was carried out with a PT1000 instrument to test melting and crystallization temperatures, with the temperature ranging from 30 °C to 200 °C and a heating rate of 10 °C/min.

Mechanical properties were tested in accordance with ASTM standards. Before mechanical testing, all specimens were conditioned at 23 ± 2 °C and 50 ± 5% relative humidity for at least 24 h. Tensile performance was measured by a WDW-20 universal testing machine following ASTM D638 [47] using Type I specimens, with a specimen thickness of 4 mm, width of 10 mm and tensile speed of 100 mm/min. Notched Izod impact strength was tested using a PIT501J-2 pendulum impact tester according to ASTM D256 [48]. Flexural properties were determined via a three-point bending test on the same universal testing machine according to ASTM D790 [49] at a loading rate of 2 mm/min and support span of 64 mm. Five specimens were tested for each group, and the results were reported as mean ± standard deviation.

## 4. Conclusions

In this study, CF-Cu-BTC/LDPE composites were prepared by introducing Cu-BTC-modified CF into an LDPE matrix through melt blending. The Cu-BTC coating anchored on CF strengthens the interfacial interaction between the fiber and the polymer matrix, optimizes load transfer and improves the comprehensive properties of composites. Thermal analysis showed that CF-Cu-BTC promoted the crystallization of LDPE, while TG results confirmed the stable incorporation of the modified fibers. Among all samples, M_4_ with 20 wt% CF-Cu-BTC exhibited the best mechanical performance, with tensile and flexural strengths of 26.63 MPa and 25.15 MPa, respectively. In conclusion, this study confirms that Cu-BTC interfacial modification is an effective strategy to enhance the fiber/matrix interaction and mechanical properties of CF-reinforced LDPE composites. The prepared composites exhibit promising application prospects in lightweight structural components, automotive parts, and engineering plastic products.

## Figures and Tables

**Table 1:** CF-Cu-BTC/LDPE composite blending scheme.

	LDPE (wt%)	CF-Cu-BTC (wt%)	CF (wt%)
M_1_	100	0	0
M_2_	95	5	0
M_3_	90	10	0
M_4_	80	20	0
M_5_	70	30	0
CF/LDPE-20	80	0	20

## Data Availability

The data that support the findings of this study are available on request from the corresponding author.

## Supplementary Materials

The following supporting information can be downloaded at: https://www.mdpi.com/article/10.3390/molecules31152573/s1. Table S1. DSC parameters of LDPE and CF-Cu-BTC/LDPE composites; Table S2. TG thermal decomposition parameters of LDPE and CF-Cu-BTC/LDPE composites; Table S3. Tensile strength of reported carbon fiber/polyethylene composites; Figure S1. Tensile modulus and elongation at break of LDPE and CF-Cu-BTC/LDPE composites.
