# Supplementary material for: Effect of Cu-BTC-Modified Carbon Fiber on Interfacial and Mechanical Properties of Polyethylene Matrix Composites
Source: Molecules. 2026 Jul 23;31(15):2573. doi: 10.3390/molecules31152573 (PMC13467932; doi:10.3390/molecules31152573)

Effect of Cu-BTC-Modified Carbon Fiber on Interfacial and Mechanical Properties of Polyethylene Matrix Composites

Shuzhen Guo [1], Shanshan Xu [1,2,*] and Yuhao Ma [2,*]

[1] Shandong Fangda New Material Technology Co., Ltd., Zibo 255049, China
[2] School of Materials Science and Engineering, Shandong University of Technology, Zibo 255049, China
* Correspondence: xushanshan@sdut.edu.cn (S.X.); m17796658607@163.com (Y.M.)

Table S1. DSC parameters of LDPE and CF-Cu-BTC/LDPE composites.

Sample	T_m/°C	T_c/°C	ΔH_m/J·g^{-1}	ΔH_c/J·g^{-1}	X_c/%
M_1	123.12	107.34	145.30	135.00	49.59
M_2	125.70	114.19	103.91	108.25	37.33
M_3	125.19	114.31	104.94	58.26	39.79
M_4	126.63	114.77	90.23	67.48	38.49
M_5	127.95	114.55	66.21	76.94	32.28

Table S2. TG thermal decomposition parameters of LDPE and CF-Cu-BTC/LDPE composites.

Sample	T_{onset} (°C)	$T_{5\%}$ (°C)	T_{max} (°C)	Residue (%)
M_1	461.26	450.18	481.68	~0
M_2	453.76	434.33	478.47	2.29
M_3	456.10	431.51	479.76	7.37
M_4	458.10	446.53	480.9	19.99
M_5	456.84	439.51	479.91	28.43

Table S3. Tensile strength of reported carbon fiber/polyethylene composites

References	Matrix	Tensile strength (MPa)
Ref.1	Polyethylene	19.12
Ref.21	Polyethylene	27.4
Ref.44	Polyethylene	21.5
Ref.45	Polyethylene	23.1
Ref.46	High-density polyethylene	15.15
This work	Polyethylene	26.63

Figure S1. Tensile modulus and elongation at break of LDPE and CF-Cu-BTC/LDPE composites.

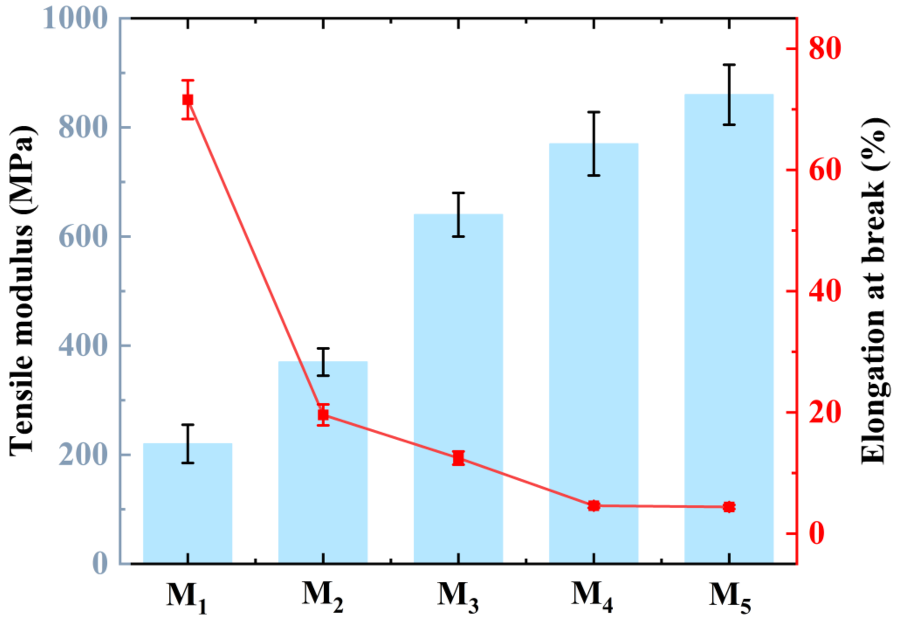

Supplement: Supplementary file 1 [file molecules-31-02573-s001.zip › molecules-4401484-supplementary.pdf]
